# Automated Flushing System for Post-Processing in Microfluidic Device Fabrication

**DOI:** 10.3390/mi17050538

**Published:** 2026-04-28

**Authors:** Sebastian Zapata, Brady Goenner, Dallin S. Miner, Bruce K. Gale, Gregory P. Nordin

**Affiliations:** 1 Electrical and Computer Engineering Department, Brigham Young University, Provo, UT 84602, USA; 2 Mechanical Engineering Department, University of Utah, Salt Lake City, UT 84112, USAbruce.gale@utah.edu (B.K.G.)

**Keywords:** microfluidics, DLP-SLA 3D printing, automated post-processing, resin flushing, chip-to-chip interconnect, pneumatic control system, fluidic circuit model, microfluidic valves

## Abstract

Post-processing remains a major bottleneck in the fabrication of microfluidic devices using Digital Light Processing Stereolithography (DLP-SLA) 3D printing, where unpolymerized resin trapped within internal structures must be removed without damaging delicate features such as thin membranes, valves, and pumps. Manual flushing is slow, inconsistent, and prone to structural failure, especially as device complexity and port counts increase. Here, we present the first fully automated flushing system for DLP-SLA microfluidic devices, enabled by a standardized chip-to-chip (C2C) interconnect architecture and an electronically controlled pneumatic routing platform. A reusable 32-port flushing interface chip provides alignment, sealing, and modular coupling to arbitrary device chips through integrated microgaskets, while a network of electronic pressure controllers, differential pressure sensors, and multi-port rotary valves enable precise, programmable application of pressure, vacuum, and solvent conditions. We introduce a fluidic-circuit model of the system that relates applied pressure to the pressure drop across device structures and experimentally validate this model using channels with varying fluidic resistances. Using this platform, we demonstrate robust flushing of both passive (straight and serpentine channels) and active (valves, pumps) microfluidic elements, as well as application-specific devices including mixers and concentration-gradient generators. Our system eliminates manual handling, improves valve membrane survival, and provides repeatable flushing across a broad range of device geometries. This work establishes a scalable foundation for automated post-processing in 3D-printed microfluidics and significantly advances the practicality of DLP-SLA fabrication for complex, multi-layered microfluidic devices.

## 1. Introduction

In analytical chemistry and bioassays, microfluidic devices can dramatically reduce reagent consumption, waste production, and overall assay costs while enabling high-throughput, precise fluid manipulation [1]. However, traditional fabrication methods such as soft lithography remain time-intensive and expensive for rapid prototyping, particularly for complex three-dimensional geometries. Digital Light Processing Stereolithography (DLP-SLA) has emerged as a promising alternative, offering rapid turnaround, low cost, and high resolution suitable for microfluidic applications [2,3,4,5,6,7,8,9,10]. By leveraging the capabilities of custom 3D printers published in our earlier work [11,12], a wide variety of microfluidic devices can be rapidly fabricated (~20 min for most designs) and with excellent resolution, featuring internal components as small as a few pixels wide (~15 µm).

The workflow for DLP-SLA 3D printing consists of sequentially creating layers of polymerized resin on a build platform mounted on a vertical stage by projecting images with a UV light engine that define the polymerization pattern for each layer [11]. This UV exposure triggers monomer crosslinking, resulting in solid polymerized material. The projected images are generated by processing the 3D model of the desired device with a slicer tool to generate vertical increments representing the XY features at a given height. Consequently, a device resulting from this fabrication process consists of a partially polymerized block of resin comprising stacked layers with various patterns that define voids (negative features) within it.

Despite the advantages of DLP-SLA fabrication, devices produced with this method are not immediately operational. In particular, post-processing steps needed for taking the device to an operational state—especially the removal of uncured resin from internal features—often require manual intervention and can compromise device integrity. These post-processing requirements introduce reliability and throughput limitations to fabrication and can increasingly dominate the workflow as device complexity scales.

This work addresses these limitations by presenting a system designed to automate the removal of uncured resin from negative features in the post-processing stage of device fabrication. By automating this critical post-processing step, which is the primary remaining manual bottleneck in DLP-SLA microfluidic fabrication, our system enables research laboratories to achieve substantially higher device yield and repeatability without requiring full industrial-scale automation or changes to existing printing workflows. We start by describing the process of manual post-processing along with its challenges, build upon previous developments that the system relies on, introduce the system architecture with a brief characterization of its operation, and finally show the system performance on a wide variety of microfluidic structures.

## 2. Post-Processing in DLP-SLA Microfluidic Fabrication

A device fabricated using DLP-SLA is taken through a post-processing routine to reach an operational state [13]. This post-processing stage consists of two parts: (i) flushing, and (ii) UV curing. Flushing removes unpolymerized resin from negative features within the microfluidic device by applying environmental conditions at opposite ends of the features to generate a pressure difference across the functional path. This pressure difference results in a directed flow that displaces the unpolymerized resin out of the device.

This process is commonly done by manually inserting the tip of a pressure or vacuum source into access points (pinholes) that provide access to the internal structures of the device. A subsequent, but optional, step is rinsing, which is done by inserting a syringe filled with a solvent—usually isopropyl alcohol (IPA)—into one of the access points of the device and pushing the solvent through the internal structures to dissolve and remove residual unpolymerized resin.

**Figure 1:**
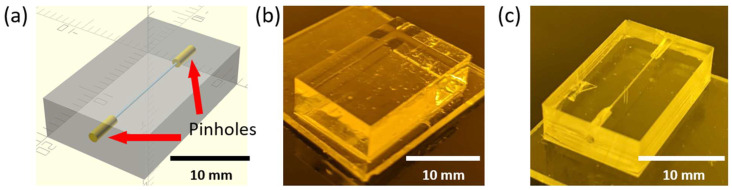
Example of a simple microfluidic device featuring a single channel going across its body. (**a**) 3D model of the fabricated device. (**b**) 3D-printed device prior to flushing. No internal structures can be seen as they are full of unpolymerized resin. (**c**) Flushed device. All the unpolymerized resin has been removed and the internal structures can be seen through the polymerized material.

One of the simplest examples of a microfluidic device fabricated using DLP-SLA 3D printing is shown in Figure 1, which depicts a polymerized block containing a single horizontal channel connected to the outside through pinholes on each end. These pinholes are wider apertures on the side walls of the device intended for Polytetrafluoroethylene (PTFE) tubing to be inserted to connect the features embedded in the device to external lab equipment, such as pressure sources, solenoid valves, or syringes. This configuration provides a straightforward chip-to-world interface. An example of a flushed device comprising a single channel is shown in Figure 1c, where the internal structures become visible once the unpolymerized resin has been removed from the unflushed device in Figure 1b.

Following flushing, UV curing is performed by placing the device inside a curing chamber under UV light for approximately 20 min. This step increases the degree of crosslinking, transforming the partially polymerized structure into a mechanically robust device suitable for operation.

The manual nature of the flushing task gives rise to several issues in the post-processing stage of fabrication that can decrease its efficiency. First, immediately after being printed, a device will only be partially polymerized, which results in a relatively soft print prone to damage with repeated insertions and removals of the tip of a pump or syringe in the pinholes of the device. Variability in operator dexterity increases the likelihood of damaged or broken structures in the process. Second, the amount of time required to sequentially insert and remove the tip of a pump or syringe by hand from the pinholes of the device is directly proportional to its complexity (since the number of pinholes needed increases), adding significant overhead to the overall fabrication process as more components are added to the design. This also increases the likelihood of damaging the device as explained earlier. Finally, the lack of fine control over the pressure levels being applied with the pump or syringe makes it so that delicate structures such as membranes, which are frequently used in components such as on-chip valves and pumps, may be damaged or broken in the process.

Collectively, all these factors introduce failure modes in the post-processing stage of fabrication that decrease its efficiency, scalability, and reproducibility. Mitigating such failure modes is the motivation for the development of a platform to automate the flushing stage in microfluidic device fabrication, which is presented in the following sections.

## 3. Component Routing Scenarios

Several aspects of the design of a microfluidic device need to be taken into consideration for successful flushing. Natural constraints arise on the minimum feasible dimensions of the components in a device, such as the width and height of the cross-section of channels [11], which are a function of both hardware limitations as well as optical properties of the printer and the resin formulation. These have been expounded upon in previous work [12,14].

Additionally, certain guidelines should be followed to guarantee an effective path for removing unpolymerized resin from the internal structures. It should be noted that the following guidelines do not attempt to provide an exhaustive treatment for prediction of flushable paths but rather provide general rules to guarantee “flushability” in the most general cases. Furthermore, they do not address structure-specific considerations (some of which are dealt with in a later section of this work), rather, they are focused on the general layout of components disregarding their potential internal requirements.

**Figure 2:**
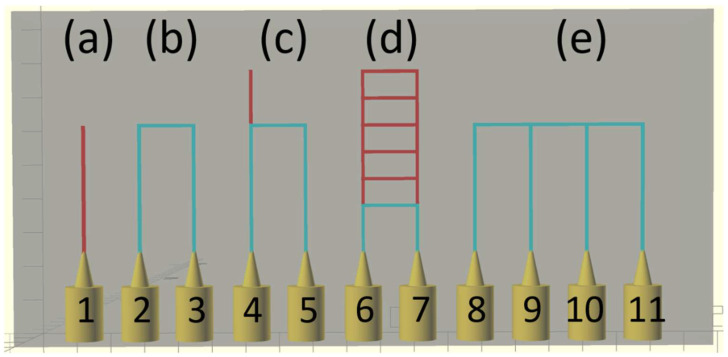
Basic scenarios for microfluidic routing. Cases (**a**–**e**) are described individually in Table 1. Channels shown in cyan are viable flow paths for post-print flushing while channels shown in red are unviable or problematic paths.

As shown in Figure 2, multiple combinations can be chosen for routing structures within the design; however, certain choices will not guarantee successful flushing. Table 1 describes the different cases and their specific considerations:

## 4. Chip-to-Chip (C2C) Interconnects

Another aspect to consider when flushing a microfluidic device is that flushing can be done by applying the pressure difference through pinholes embedded in the device as shown in Figure 1 and Figure 2, or it can be done using chip-to-chip interconnects.

**Figure 3:**
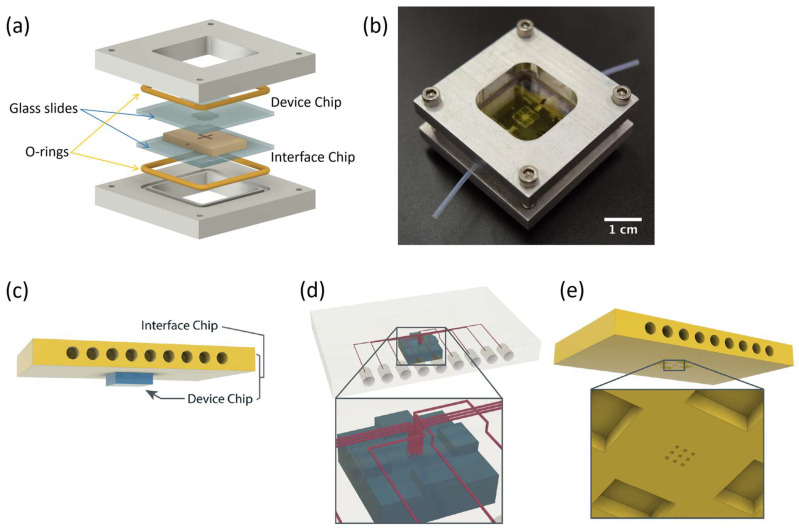
Major parts required for setting up an interface between two distinct microfluidic devices using interconnects. (**a**) Clamping mechanism for interface and device chips. (**b**) Photo of clamped interface and device chips ready for operation. (**c**) Illustration of connected interface chip and device chip (clamping mechanism omitted). (**d**) Illustration of the interior of the interface chip, showing the routing from the device chip to the pinholes. It also shows the alignment blocks on the surface of the device chip (in blue) that fit into the interface chip. (**e**) Underside of the interface chip and interface closeup, showing the corresponding recesses for the alignment blocks present in the device chip. It also shows the array of channel terminations ending on the flat bottom surface of the chip. After Ref. [15].

In previous work [15], we developed 3D-printed chip-to-chip microfluidic interconnects, where a world-to-chip “interface chip” was designed to route up to hundreds of connections from a smaller “device chip” to larger lab equipment. In this sense, the interface chip serves as a routing point with connections going from the device chip to larger pinholes for PTFE tubing, and the smaller device chip encapsulates all the functionality needed for the given microfluidic application. The interface chip is aligned and clamped together with the device chip using a custom aluminum clamp as shown in Figure 3a,b.

Figure 3c,d show how the proper placement of both interface and device chips creates an effective routing from the structures in the device chip to the pinholes in the interface chip. Matching features are placed on both chips to ensure proper horizontal alignment as shown in Figure 3d,e.

**Figure 4:**
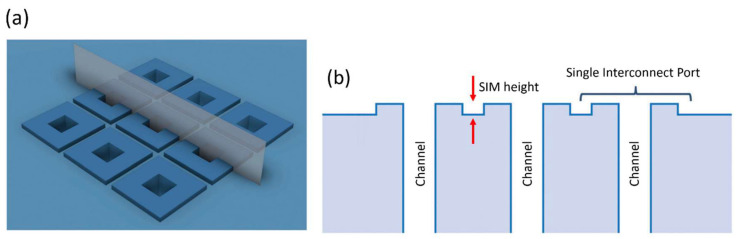
Microgaskets general architecture. (**a**) Illustration of an array of microgaskets (SIMs). Taken from Ref. [15]. (**b**) Cross-sectional profile of an array of SIMs. Taken from Ref. [15].

Another important feature previously developed is that of Simple Integrated Microgaskets (SIM) [15], which are mounted on the terminals of the channels in the device chip. When the device chip is clamped to the interface chip, the corresponding channel terminals in the interface chip are pressed against the microgaskets, compressing them, and establishing a leak-free interconnection. Figure 4a shows the architecture of an array of microgaskets with a cross-sectional profile shown in Figure 4b, which highlights the protrusion of the microgaskets above the contact surface of the device chip. We typically use a microgasket height of 10 µm.

**Figure 5:**
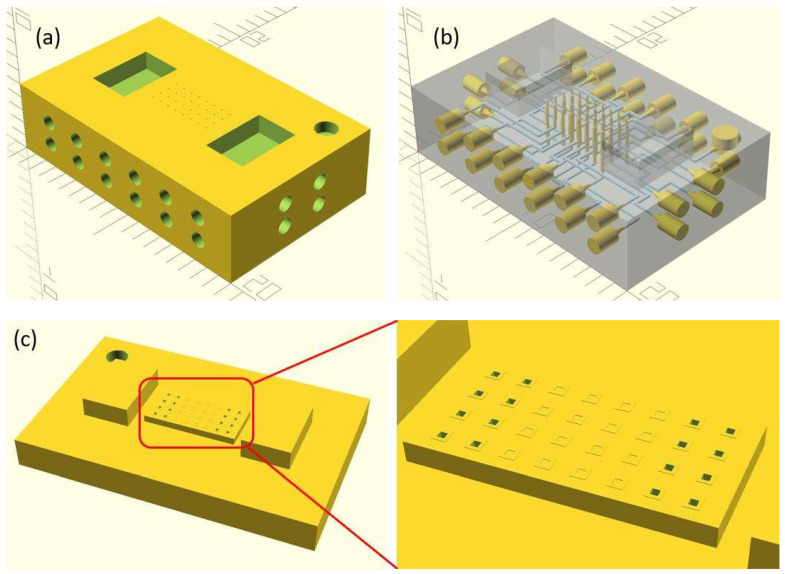
(**a**) Illustration of the standard interface chip, displaying an array of connections on the top along with recesses for alignment blocks. It also shows the pinholes surrounding the sides for PTFE tubing insertion. (**b**) Interface chip in transparency mode, showing the routing between the top surface array and the side pinholes. (**c**) Arbitrary device chip conforming to the standard interconnect structure of the system, with corresponding alignment blocks and an array of microgaskets, mounted on a pedestal. The closeup on the right shows the array of microgaskets. This example also shows that though 32 interconnects are available, not all of them need to be employed, with the example showing only 16 in use (open) due to specific design choices for this particular device.

In this work, we employ these interconnect capabilities to develop a standardized interface chip to provide a connection between an automated system that will generate the controlled environmental conditions necessary for flushing and an unflushed microfluidic device (device chip). Figure 5a shows an illustration of the standard interface chip developed for this work, which is used for flushing a device chip, and Figure 5b shows its inside with corresponding routing from the pinholes to the array of channel terminations. Figure 5c shows an arbitrary device chip that conforms to the standard features of the interface chip. This creates a set of standard design rules that allow the user to connect any device chip to the system regardless of the combinations of internal structures needed for the specific microfluidic application, so long as it conforms to the hardware constraints of the system, which will be discussed in the Section 5.

A notable feature shown in Figure 5c is the inclusion of a central pedestal as a platform for the array of microgaskets in the device chip. This is necessary to mitigate issues that arise from the manual nature of planarization of the 3D printer prior to printing a device, where the platform on which the device is fabricated needs to be aligned with the focal plane of the light engine that projects images for each layer of the device. If this planarization routine is less than ideal, slight plane misalignments occur that reduce the equal distribution of exposure across the surface of a layer, inducing a slight—and nearly unobservable—vertical curvature on one of the corners of the fabricated device, which bends such corner upward.

**Figure 6:**
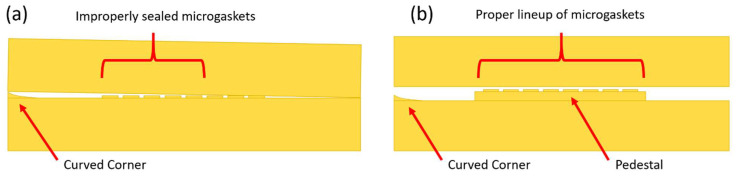
Vertical alignment artifact between interfacing devices introduced by curvature in a corner, which results after poor planarization of the 3D printer. (**a**) Vertical cross-section of the interface chip (top) and device chip (bottom) stack, showing vertical misalignment when, due to variable degrees of quality in planarization, a slight curvature is introduced in the surface of either device in a specific corner. This causes certain microgaskets to not have a proper seal with the surface of the interface chip. (**b**) Vertical cross-section showing proper alignment of interface and device chips as the array of interconnects is mounted on a pedestal not susceptible to the corner curvature induced by the variable quality of planarization of the printer used for fabrication. Sizes of microgaskets and curved corners are not to scale; larger dimensions were used for illustration purposes.

Even though this curvature is minimal and only seen on a specific corner of the device, since the microgaskets are only 10 µm tall, such a curvature can be big enough to prevent some of them from having full contact with the surface of the interface chip, as shown in Figure 6a. Hence, they are mounted on a pedestal, which should be designed to be tall enough to keep them isolated from the effects of potentially curved corners. This ensures proper contact between the microgaskets and the bottom surface of the interface chip, as shown in Figure 6b. Though there can be significant variability from one device to another in the curvature induced by the artifact described above, a pedestal of at least 70 layers (700 µm) was observed to offer a definitive solution to this issue.

It should be noted that certain microfluidic structures require different routing schemes to the externally imposed pressure conditions for the various inputs/outputs depending on whether these structures are being flushed or operated. This is the case for any active components such as valves and pumps, as opposed to passive components like channels or reservoirs. As shown in previous work [10], both an inlet and an exit channel are needed for flushing the control chambers of valves, but only one of them is needed to operate it. In order to account for that, an interface chip suited for flushing the device will allow pathways for both inlet and outlet pneumatic channels, but the interface chip suited for operating the device will block one of those channels and use the other one as the pathway for applying the pressure needed to actuate the component. Therefore, to make the distinction between interface chips with routing suited for flushing versus routing suited for operation of a device, we will refer to them as the “flushing” interface and “operational” interface chips, respectively. In sum, the only difference between both kinds of interface chips is that for operational interface chips, certain connections that were used for flushing will be blocked, thus allowing the control structures (such as pneumatic chambers in valves or pumps) to be operated with a single channel.

Additionally, the distinction made above between flushing and operational interface chips imposes the requirement that whenever users design a new device chip to be flushed with the system, they should also design an accompanying operational interface chip since the flushing interface might not match the specific needs for operation. More importantly, as will be shown later, the flushing interface chip is meant to be a reusable device permanently attached to the system with PTFE tubing, so operation of a flushed and cured device will take place outside the working environment for flushing, thus requiring a separate device to operate it.

The requirement of operating a device chip with a separate operational interface chip, distinct from the flushing interface chip, may be deemed undesirable at first, as it potentially necessitates additional design work for an operational interface chip with every device chip iteration during the prototyping phase. However, when considering the fabrication of microfluidic devices at a large scale, the use of interface chips for both flushing and operation becomes increasingly attractive once a definitive device chip design is developed. For example, a lab may print dozens or even hundreds of replicas of a device and use a single flushing interface chip for post-processing, and a single operational interface chip for operating devices one after another. This significantly reduces the overhead of having to attach permanent pieces of PTFE tubing to each device chip to connect them to external equipment for operation, and instead PTFE tubing is attached to a single operational interface chip so that it can be connected to external equipment. This operational interface chip can then be repeatedly clamped to every device chip that needs to be operated using the clamping mechanism described above. This eliminates both material waste and time required to attach tubes to each device chip, thus significantly expanding the efficiency of microfluidic device fabrication and operation at a large scale.

## 5. Tools and Equipment

Having established a framework for coupling an arbitrary device chip to a static system through the use of chip-to-chip interconnects present in a flushing interface chip, a necessary set of tools and equipment need to be defined to provide environmental conditions suitable for flushing the device chip in an automated fashion.

**Figure 7:**
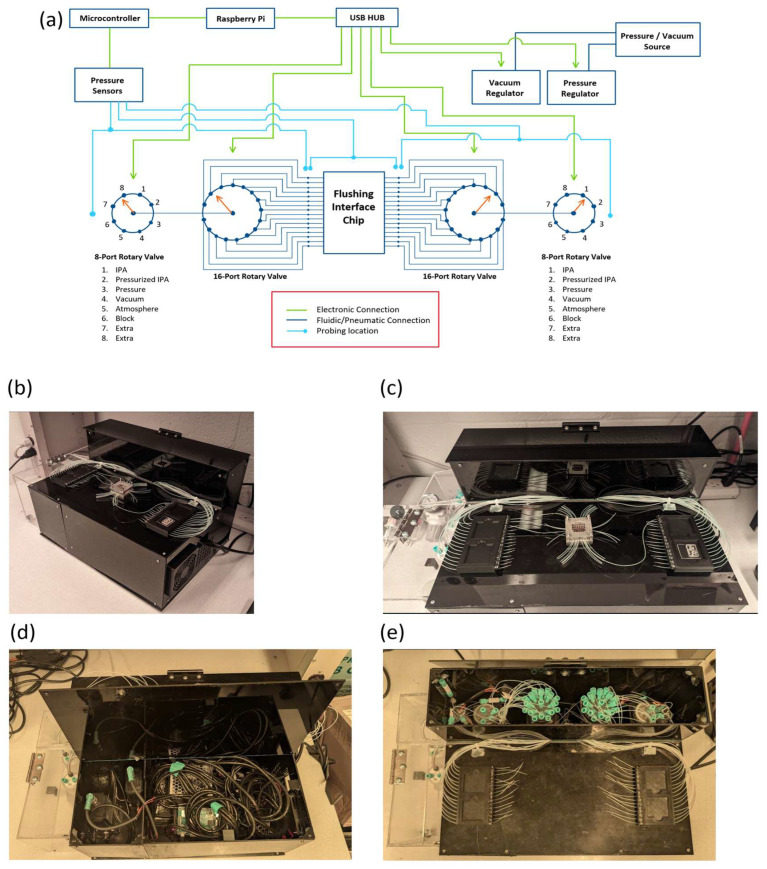
(**a**) System diagram depicting all electronic and pneumatic components employed to execute and monitor automated flushing of microfluidic devices through a flushing interface chip. (**b**) Physical implementation of the system. (**c**) Closeup of the system with the PTFE tubing coming from the 16-port ERVs in the inside of the system box to the flushing interface chip clamped to a sample device chip. (**d**,**e**) The arrangement of electronics inside the system, including the Raspberry Pi computer, ERVs, and power supplies, among others.

Figure 7a shows a diagram of the full system developed, where (starting from top to bottom) a Raspberry Pi was used to control the different electronics present in the system and to provide a user interface through a locally hosted web app. A microcontroller (Teensy 3.2, Digikey Inc., Thief River Falls, MN, USA) was connected to differential pressure sensors (Honeywell 26PCFFA6G, Digikey Inc., Thief River Falls, MN, USA) mounted at strategic locations of the system to monitor the pressure at different points in the flushing routine. Pressure and vacuum sources were connected to electronic pressure controllers (EPCs) (SMC ITV2090-RCN2BS, SMC Pneumatics, San Jose, CA, USA) to provide desired pressure and vacuum levels for a variety of user-defined environmental conditions (air, IPA, waste, etc.). Finally, a set of electrical rotary valves (ERVs) were laid out to both select the appropriate environmental condition as well as to select the right port in the flushing interface chip to which to apply the environmental condition selected. As observed in Figure 7a, there are two 8-port ERVs (Aurora Pro Scientific, Midland Park, NJ, USA) on either side of the system which enable the selection of arbitrary environmental conditions, such as pressurized air, pressurized water, vacuum, etc., on both sides of the flushing interface chip. Subsequently, their outputs feed into the input of a pair of 16-port ERVs (Aurora Pro Scientific, Midland Park, NJ, USA) that route the selected environmental conditions to single ports on each side of the flushing interface chip.

The setup described above allows the operator to arbitrarily apply environmental conditions with certain limitations, which must be kept in mind to properly operate the system. First, only one port on each side of the flushing interface chip can be selected at a time, which makes the execution of the flushing routine a sequential process of selecting one effective path for flushing per actuation. Second, since there are only 16 ports on either side of the flushing interface chip, there are up to 32 ports that can be laid out for flushing purposes in the device chip, with the requirement that flow will only be feasible when the inlet and exit channels to the structure being flushed are routed to opposite 16-port ERVs. If this requirement is not met, the same environmental condition will be applied to both ends of the structure and no flow will be induced, making flushing of unpolymerized resin impossible. Finally, since the connection between 16-port ERVs and the pinholes in the flushing interface chip are arbitrary, the chip designer must arrange the routing of connections within the device chip to conform to the correspondence of ERV ports to flushing interface chip ports. Both decisions are up to the chip designer but must be consistent with each other so that proper routing of externally imposed pressure conditions can be ensured.

With proper design and implementation, the only requirements for the automated flushing system to work are providing a pressure and vacuum supply, electric power, and an internet connection for connecting to the hosted web app that allows the control of the system. Figure 7b–e show the implemented system and basic layout of connecting PTFE tubing where, upon clamping the device chip to the flushing interface chip, the flushing routine can be executed with no need for insertions and removals of hardware in and out of the device being flushed. Full control of pressure and vacuum levels can subsequently be applied. These features aim to solve the issues that arise from manual flushing, as was pointed out earlier in this work.

## 6. System Characterization and Flushing Process Analysis

To better understand the dynamics of unpolymerized resin removal within our system, we first develop a simplified model that captures the dominant physical interactions and identifies figures of merit that determine flushing performance. Then we validate the model by observing pressure drops across the different sections of the system and corroborating their quantitative relationships. This will serve the purpose of laying out a foundational understanding for monitoring the flushing of specific devices—such as the ones shown in the Section 7—as well as scaling the automation capabilities of our system in future work. We note that all devices fabricated as part of the study reported in this paper use the same resin formulation introduced in Ref. [11] and used in Refs. [12,15]. It comprises poly(ethylene glycol) diacrylate (PEGDA, MW258) with a 1% (*w*/*w*) phenylbis(2,4,6-trimethylbenzoyl)phosphine oxide (Irgacure 819) photoinitiator and a 2% (*w*/*w*) 2-nitrophenyl phenyl sulfide (NPS) UV absorber.

To perform the analysis introduced above, we draw on the well-established hydraulic–electric analogy, in which microfluidic networks are represented using concepts from circuit theory. This analogy has been widely used to describe steady microfluidic flows, derive hydrodynamic relationships, and analyze network behavior in terms of pressure sources and flow resistances [16,17,18,19,20]. In this framework, our flushing system consists primarily of pressure sources and fluidic resistors, which correspond directly to voltage sources and electrical resistors in circuit theory.

**Figure 8:**
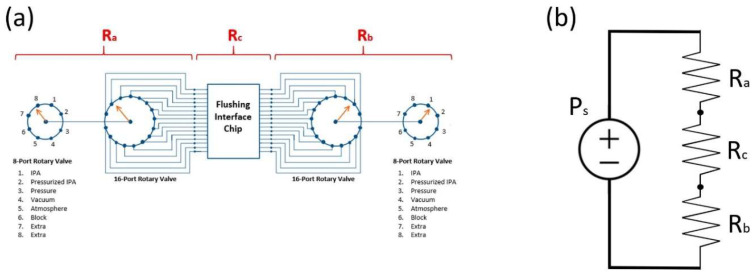
(**a**) Fluidic portion of the system with markers for the corresponding inherent fluidic resistances of each section. (**b**) Analogous system model created using circuit theory concepts.

To apply this analogy, we divided the fluidic portion of the system into three main sections, as shown in Figure 8a, delimited by the location of the pressure sensor probes (see Figure 7a). This effectively created three main sections with inherent fluidic resistances denoted by R_a_, R_b_, and R_c_. In that sense, R_a_ and R_b_ correspond to the fluidic resistance created by the combination of actuated ERVs and connecting PTFE tubing in their respective sections of the system. Additionally, R_c_ corresponds to the compound fluidic resistance of the flushing interface chip and the mounted device chip combined, with the former being constant since it is permanently attached to the system, and the latter depending on the fluidic resistance of the given design. By applying different pressure conditions at both ends of the system, such as pressurized air on one end and vacuum on the other, the net effect is a source pressure difference, which is denoted as P_s_, and is applied globally to all three main sections in the series. With these parameters, namely, R_a_, R_b_, R_c_, and P_s_, we can establish an analogous representation using circuit analysis techniques and model the system as a simple circuit with a voltage supply (pressure difference) and three resistors (microfluidic resistors) in the series as depicted in Figure 8b.

With a circuit representation of our system, we can derive some useful relationships between the applied pressure (Ps) and the pressure drop measured across each fluidic resistor by making certain assumptions about the components in Figure 8b. Since the system is relatively symmetric (with variability introduced by small differences in the length of PTFE tubing used for connecting some of the components in either side), we can assume that R_a_ ≈ R_b_. Moreover, since for the purpose of analysis we only care about R_c_ and its relationship to the fluidic resistance of the rest of the system, we shall define the fluidic resistance of the rest of the flushing system as R_sys_ = R_a_ + R_b_. It should also be noted that regardless of the value of R_c_ with respect to R_sys_, its value can be expressed as R_c_ = αR_sys_, where α expresses the proportional relationship between them and has a value within the open interval (0, ∞). These statements along with simple circuit analysis for a voltage divider enable us to derive an expression for the pressure drop across R_c_ (denoted as ∆P_c_) as follows:

(1) $$∆P_{c}\;=\;(\frac{\alpha }{\alpha +1})Ps$$

Equation (1) lets us consider three main cases that relate the steady-state pressure drop across R_c_ (∆P_c_) to the source pressure applied across the system (P_s_) and the ratio of fluidic resistance between R_c_ and the rest of the system (R_sys_) as follows:

✓ Case 1: R_c_ >> R_sys_. The fluidic resistance of the R_c_ block is significantly higher than that of the system (α >> 1). Equation (1) yields:

∆P_c_ ≈ P_s_

✓ Case 2: R_c_ << R_sys_. The fluidic resistance of the R_c_ block is significantly lower than that of the system (α → 0). Equation (1) yields:

∆P_c_ ≈ 0

✓ Case 3: R_c_ ≈ R_sys_. The fluidic resistance of the R_c_ block is similar to that of the system (α~1). Equation (1) yields:

∆P_c_ = *c*P_s_

This analysis shows cases 1 and 2 as particular cases of the more general situation depicted in case 3, where *c* is the proportionality ratio between ∆P_c_ and P_s_, and its value lies in the open interval (0, ∞).

**Figure 9:**
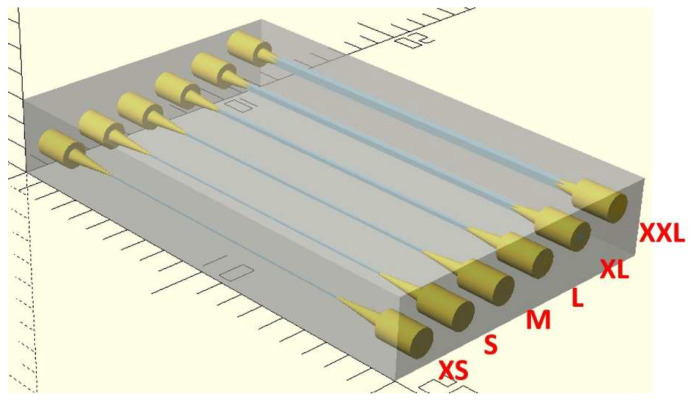
Device for fluidic resistance testing in transparent mode, showing labeled straight channels of different cross-sectional dimensions connected to the outside by corresponding pinholes, suited for pressure drop observations.

To illustrate these relationships, the device in Figure 9 was designed as a proxy for the clamped flushing interface and device chips to represent their compound fluidic resistance at different values (R_c_). This design contains a series of six straight channels of varying cross-sectional dimensions, resulting in different values of fluidic resistances for each of them, which translates into different values for the α coefficient from Equation (1). This will effectively change the behavior of the steady-state pressure drop across R_c_ after flushing, exemplifying the derivations described in cases 1, 2, and 3.

Steady-state pressure drop was measured sequentially on each of the channels in the device shown in Figure 9 (each in turn representing R_c_ in Figure 8) as well as on the surrounding sections of the system (represented by R_a_ and R_b_ in Figure 8) while pressurized air (P_s_) was applied across the system. Pressurized air at 10 PSI was applied since it is a common pressure level used for successful flushing given our resin and device features, and it is about the same pressure exerted when moderately pushing air through a typical lab syringe by hand. However, any combination of pressure conditions that result in a directed flow of unpolymerized resin to flush the device would work. If the applied pressure level is too low, the fluidic resistance resulting from resin viscosity and path geometry will be such that no net flow is induced. This “threshold” pressure value will vary for every device design and resin chemistry, and any pressure above this threshold value will suffice for successful flushing.

**Figure 10:**
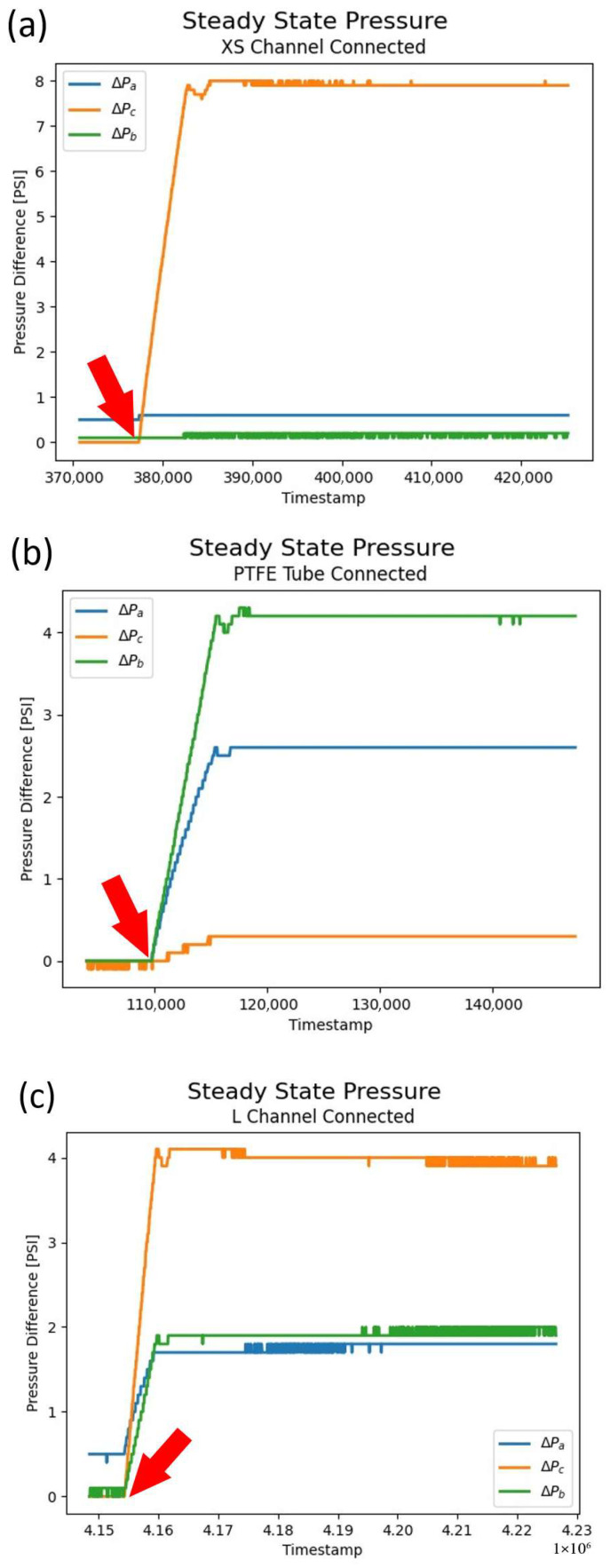
Data collected from measuring pressure drop across the selected channels from the device in Figure 9 and PTFE tubing (each in turn representing R_c_) and the other sections of the system (representing R_a_ and R_b_) while running pressurized air through the system at about 10 PSI (P_s_). (**a**) XS channel connected to the system, showcasing the situation described by case 1 from Equation (1). (**b**) PTFE tube connected to the system, showcasing the situation described by case 2 from Equation (1). (**c**) L channel connected to the system, showcasing the situation described by case 3 from Equation (1).

The measurements from two specific channels in the device shown in Figure 9 and plain PTFE tubing connected to the system were selected as they more closely resembled the three base cases for Equation (1) in terms of fluidic resistance compared to the rest of the system. The dimensions for each of them are 45 µm × 50 µm and 152 µm × 150 µm for the XS channel and L channel, respectively, and an inner diameter of 558 µm for the section of PTFE tubing that was used as well. Figure 10 shows the data collected as a result of these experiments.

The red arrow in each plot points to the instant at which the pressure difference on the system was raised from 0 to P_s_, and the subsequent pressure drop response was triggered in each of its sections. Figure 10a shows the pressure drop caused on each section when the XS channel was connected to the system, and as pointed out from case 1 of Equation (1), the XS channel pressure drop is about the value of P_s_. This is caused by the fluidic resistance of the XS channel being significantly larger than that of the rest of the system and therefore dominating the pressure drop. Figure 10b shows the converse case, where the fluidic resistance of the channel connected (PTFE tube) was significantly smaller than that of the system, causing most of the pressure drop to take place on R_a_ and R_b_ and the drop on R_c_ nearing 0 (case 2 of Equation (1)). Finally, Figure 10c shows the situation where the fluidic resistance of the system is similar to that of R_c_ (the L channel) and the pressure drop is proportional among them, with the *c* factor from case 3 of Equation (1) being shown to be about 0.5 from the plotted data (α factor being about 1).

It should be noted that ideally, after a steady state has been reached, the combined pressures of all three sections would add up to P_s_ (10 PSI in the case of our experiments); however, this is not the case in either of the three plots due to some unmonitored pressure losses in the system. These are caused by both potential pressure leaks not directly obvious while operating the system, as well as sections of the system physically out of reach of the pressure sensor probes. However, regardless of the pressure losses not accounted for in the measurements (which are constant), the trends observed in Figure 10 can be directly mapped to the 3 base cases derived from Equation (1), which validates the model built to represent the system.

The relationships analytically derived and experimentally observed in this section give further insight into how the removal of unpolymerized resin inside of microfluidic devices works. When a flushing pressure difference is initially applied, R_c_ will have a large value due to unpolymerized resin filling all of the negative space features such that essentially all of the pressure is dropped across the device. However, as unpolymerized resin is flushed out, R_c_ will drop until it reaches a steady-state value. This pattern can be seen across each of the plots in Figure 10, where the pressure drop across each section initially ramps up, but then experiences a quick drop (due to the contained fluid transitioning from unpolymerized resin to air) and finally goes up slightly, reaching a steady value as the entire flow path is uniformly filled with air as the unpolymerized resin is expelled. The transition of contained material from a highly viscous liquid (unpolymerized resin) to a gas (air) or less viscous liquid (IPA) as observed by the readings of the pressure sensors is commonly known as two-phase flow. This transient state takes place in a very short amount of time (often less than one second) and its intricacies go beyond the scope of this paper. Given the simplified model proposed above, a steady-state single-phase system is assumed.

Once unpolymerized resin has been properly removed from the internal features of a device by employing the pressurized air or vacuum environments, it is standard to switch the flushing fluid from air to IPA. IPA is used to dissolve unpolymerized resin without affecting the polymerized 3D print, facilitating easier removal of resin caught in corners and along interior feature edges. This procedure is conducted long enough so that enough IPA flows through to dissolve resin residue, which is highly soluble in IPA. Following this “rinsing”, the IPA left within the device is removed using the aforementioned pressure/vacuum environmental conditions in the system. Without IPA, such trapped residual resin remains in the device and is integrated into the body of the polymerized device during the subsequent UV curing step. Because of this, even if there are residual traces of unpolymerized resin left inside the device following a thorough flushing process with pressurized air and IPA, this residue will be integrated into the device itself. The combination of flushing and UV-curing the fabricated device ultimately renders a clean internal environment for microfluidic operation.

Note that during flushing, the change in R_c_ can be measured, noting when the pressure difference between the input and output ports becomes smaller, thereby indicating when the initial air flush has been successful. At that point the system could automatically switch flushing fluids from air to IPA. While not yet implemented, this would afford a higher degree of post-processing automation for fabrication of microfluidic devices, which would offer significant benefits for manufacturing at a large scale.

## 7. Results—Flushed Structures

Leveraging the capabilities of the system described above to automate the flushing routine allows us to post-process fabricated devices with full control of environmental conditions applied and no requirements on the dexterity of the system operator. Table 2 below shows successfully flushed devices containing several common structures used in microfluidic devices, as well as some examples of devices tailored to specific applications. The second column shows evidence of successful flushing by displaying the resulting devices post-flushing, where each of the internal structures in them is fully cleared from unpolymerized resin. It also details certain flushing considerations specific to each of them.

In order to successfully flush each of these devices with the flushing system, they were designed to be compliant with the standard features required for properly setting up the interconnects with the flushing interface chip, as described in the Tools and Equipment section. Some of those standard features are proper spacing for alignment blocks, inclusion of a pedestal with properly spaced microgaskets, and correct placement of inlet/outlet channels to microgaskets that connect to opposite-side ERVs for flow feasibility.

Proper flushing of all the structures in Table 2 considers not only successful removal of contained unpolymerized resin, but also preservation of all internal structures and successful device operation after UV curing. This included repeatedly testing flow through passive structures—such as straight and serpentine channels—and testing membrane actuations for active structures—such as valves and pumps. Thus, the functionality of both passive and active devices is ensured to be preserved throughout the post-processing routine, validating the operation of the flushing system.

Besides the target designs highlighted in Table 2, additional devices used for research projects independent of this work have been post-processed with the flushing system introduced in this paper. A total of 100+ microfluidic devices have been successfully post-processed with this system with an approximate success rate of 95%, where the only failure mode was introduced by the operator improperly clamping the device chip and the flushing interface chip. Improper clamping often damaged the device chip as it is still in a soft state before undergoing UV curing, which takes place after flushing. However, this was uncommon and was the only failure mode, it being the only manual task in the process. Additionally, the flushing system increased reproducibility of devices with active components (valves and pumps) in most cases by at least 20%, where the biggest failure mode in manual flushing was the breaking of membranes due to uncontrolled high flushing pressure. In a particular design where there were several valves and pumps, device yield increased from 10% to 80% with 25 devices post-processed using the flushing system (each containing 13 membranes in on-chip valves and pumps).

Finally, it is worth noting that though overhead processing time is increased on devices with few components (decreasing overall process efficiency) compared to manual flushing due to having to clamp device chips to a flushing interface chip, once there were three or more components needing to be flushed, efficiency usually only increased. This was especially evident on designs where, under manual flushing, the operator would normally take about 5–6 min and using the flushing system, it would take about 3 min or less, which was the case of the 4-pump device in Table 2. In some outlier cases, where there was an abundance of serpentine channels, complete unfeasibility of flushing using the manual technique was reported, but using the proposed system, flushing was feasible and took between 8 and 9 min including rinsing. This was the case of the calcium assay chip in Table 2, where its fluidic resistance profile required sustained pressure over time for flushing each of its serpentine sections, which was unreasonable to do handling a manual pressure pump, but completely feasible with the flushing system.

The examples listed in Table 2 highlight the feasibility and benefits of automating the flushing of structures. As described in Section 5, the system also has several limitations, with the most significant being that the operator can only select one pair of corresponding ERV ports at a time that route the selected environmental conditions to the flushing interface chip ports. This constrains the automated flushing routine to sequentially clear one effective flow path at a time. Since the routing of the internal structures in the design of a given microfluidic device may pose multiple symmetric or asymmetric flushing paths that need to be flushed (such as the 4-pump device in Table 2), the efficiency of the process can be expanded by increasing the number and routing of ERVs employed in the system. This may decrease or even remove the constraint of sequential flushing altogether, thus further increasing the time efficiency of the fabrication process overall.

## 8. Conclusions

This work was introduced by commenting on the necessity and dynamics of the flushing routine in the post-processing stage of the fabrication of microfluidic devices. We note how the manual nature of the common techniques currently employed to perform post-print flushing introduces failure modes that decrease the efficiency of the fabrication process. In order to mitigate these failure modes, we have proposed a system to automate the flushing of microfluidic devices and have explored its design, implementation, and performance on a variety of common as well as more application-specific designs.

We note several improvements that can be incorporated into the proposed system to further increase the throughput of the fabrication process. Nonetheless, our initial work as reported in this paper introduces a combination of technologies that facilitate the automation of the post-processing task of flushing unpolymerized resin from negative features. Furthermore, it facilitates the making of SLA-DLP 3D printing an ever more accessible and scalable technology for the fabrication of microfluidic devices.

## Figures and Tables

**Table 1:** Component routing scenarios and the consequences of each case on flushing feasibility.

Case	Description
(a)	Dead-end channel. Flushing is not feasible.
(b)	Single input single output channel. Flushing is feasible.
(c)	Single input single output channel with additional dead-end section. Partial flushing is feasible.
(d)	Multiple channels in parallel with shared single input and single output. Only one of the parallel channels has guaranteed feasible flushing.
(e)	Multiple channels in series with intermediate connections routing out. Flushing is feasible by sequentially providing a pressure difference across pairs of channels to remove unpolymerized resin from each section between pinholes 8 through 11 while blocking all other channels.

**Table 2:** Both standard components embedded in a device as well as application-specific devices were successfully flushed with the system. Corresponding illustrations of their OpenSCAD designs as well as the flushed devices post-fabrication are shown. A summary of design and flushing commentary for each device are included.

OpenSCAD Design	Flushed Device	Design & Flushing Commentary
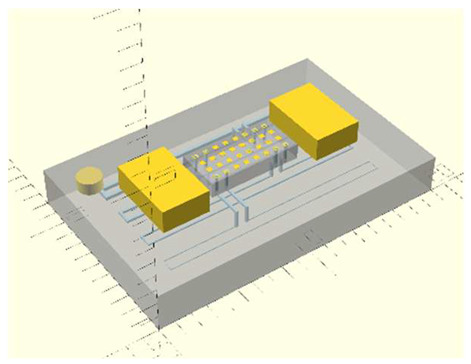	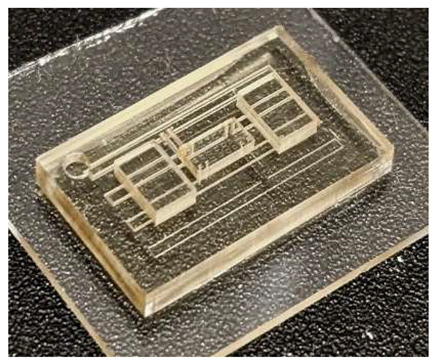	**Straight Channels Device** 4 different sizes of channel cross-sections: 45.6 µm × 50 µm, 91.2 µm × 100 µm, 152 µm × 150 µm, 228 µm × 220 µm. They were all 29,792 µm in length. The narrower and longer the channels, the more time they take to flush (more fluidic resistance). 20 channel components were fabricated and post-processed with a 100% success rate (fully flushed channels). Each of them provided reliable flow under pressures of up to 20 PSI, which is a standard pressure applied by a syringe pump. Higher pressures are supported as shown in Ref. [15].
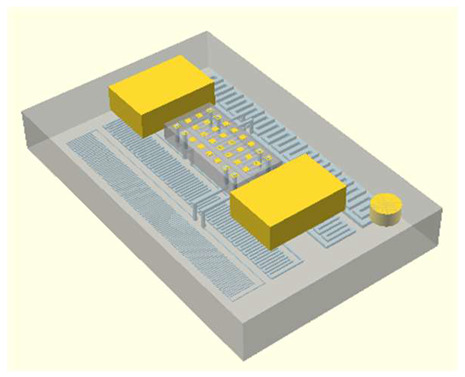	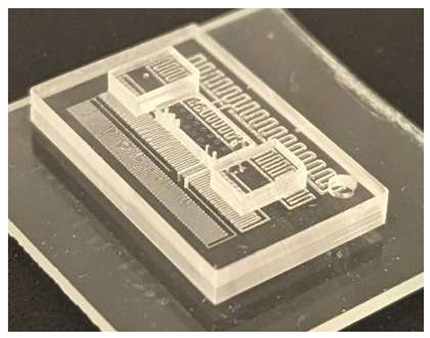	**Serpentine Channels Device** Same cross-sectional dimensions for channels as the straight channels device. Since each serpentine channel has different cross-sectional dimensions, the total length of each varies. The narrowest channel (45.6 µm × 50 µm) had a total length of 18.67 cm. It took ~8 s to flush. 20 channel components were fabricated and post-processed with a 100% success rate (fully flushed channels). Each of them provided reliable flow under pressures of up to 20 PSI, which is a standard pressure applied by a syringe pump. Higher pressures are supported as shown in Ref. [15].
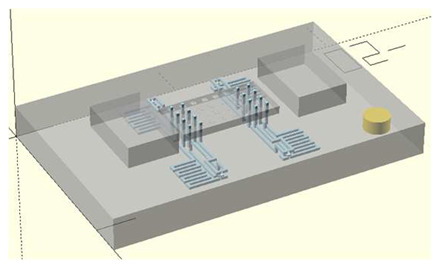 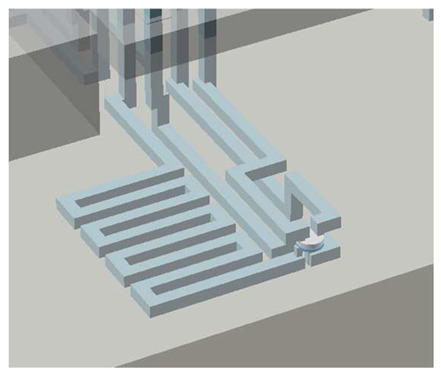	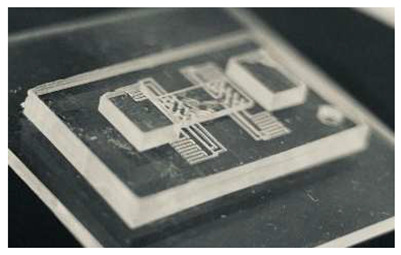 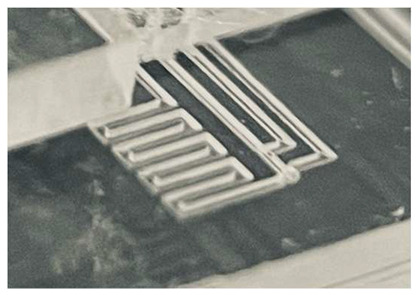	**4-Valve Device** First example of active device flushing with success rate determined by both pressure applied and membrane exposure used during printing. The design consists of 4 integrated valves with a corresponding serpentine channel in series to observe proper operation. Fine control of pressure allows for gentle flushing at the minimum pressure that induces effective flow. This lowers requirements on membrane sturdiness before flushing. Operational interface chip is required for blocking one of the channels in the control chamber for actuating each valve, as described in Section 3. 20 valves were fabricated and post-processed with a 100% success rate (fully flushed channels, control chambers, fluidic chambers, and preserved membranes). Each of them was reliably actuated over 250 times, fully blocking fluid flow when actuated.
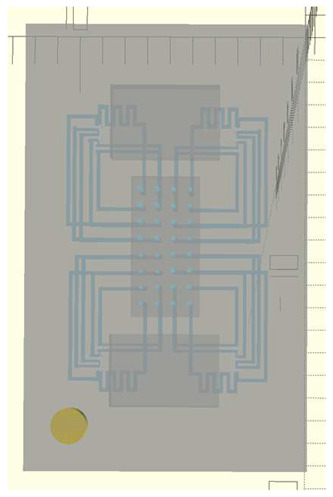 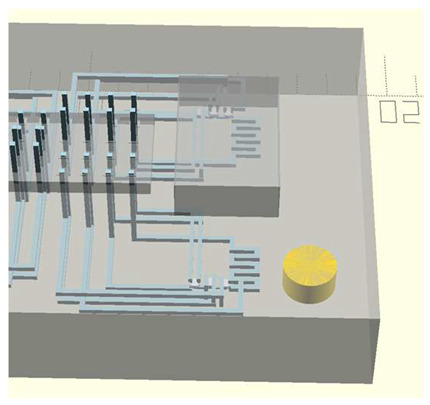	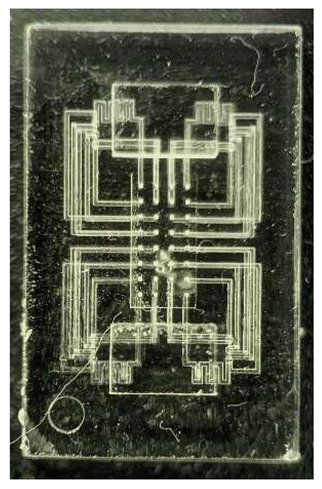 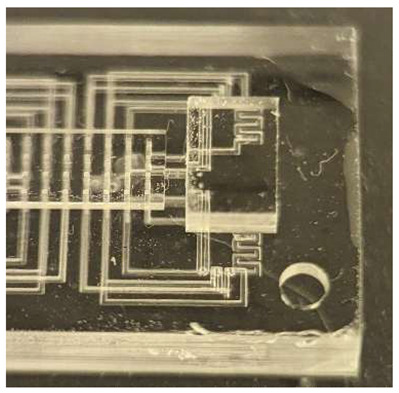	**4-Pump Device** The design includes 4 integrated pumps with corresponding serpentine channels in series to observe proper operation. Since every pump requires 8 connections for flushing, all 32 connections in the interconnect structures were used. Flushing the displacement chambers (DCs) in each pump required applying the same criterion for flushing the valves from the previous device. Fine control of pressure applied enabled membrane survival during and after flushing. Operational interface chip is also required, with higher level of complexity as the number of active components is increased. 20 pumps were fabricated and post-processed with a 100% success rate (fully flushed channels, control chambers, fluidic chambers, and preserved membranes). Each of them was successfully actuated for over a minute showcasing proper fluid pumping.
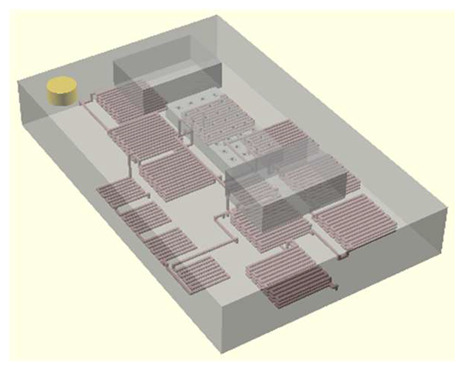	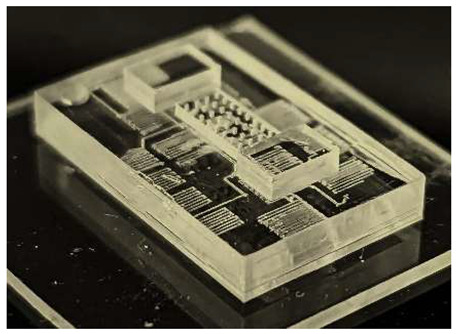	**Calcium Assay Chip** Design enables the mixing of 3 fluids with distinct mixing ratios, by adjusting the lengths of all 3 input serpentine channels. They converge into a single output serpentine channel [21]. Flushing and rinsing take about 9 min. Asymmetric design (3 inputs, 1 output) requires sequential flushing as discussed in Table 1 entry (e). Calcium absorbance assay results used in Ref. [21] are taken from calcium assay chips post-processed with the flushing system presented in this work. 20+ calcium assay chips were flushed with the flushing system with a 90% success rate. A total of 10% of the flushing attempts failed due to sections of serpentine channels being clogged during printing, which rendered them impossible to flush regardless of what mechanism was employed. This did not reflect a failure of the flushing system or its associated tools.
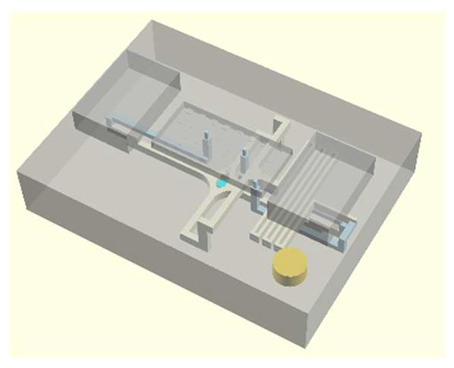	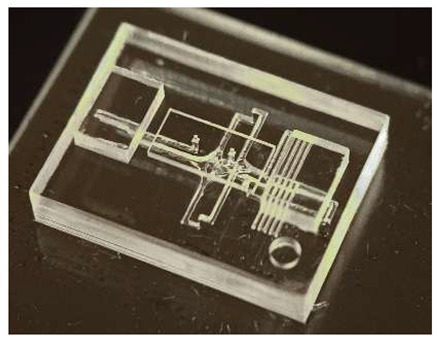	**Concentration Gradient Device** Design enables the creation of a concentration gradient in the turquoise region (center of the chip) [22]. Asymmetric design (2 inputs, 1 output) also requires sequential flushing. Though experimental results of gradient quality are beyond the scope of this work, an identical concentration gradient component (shown in teal in the OpenSCAD design) to the one used in Ref. [22] was successfully flushed 5 times with the flushing system with a 100% success rate. It reliably provided the flow path between both inputs and the output channel, showing proper flushing.

## Data Availability

The original contributions presented in this study are included in the article. Further inquiries can be directed to the corresponding author.
